# An Azole-Resistant *Candida parapsilosis* Outbreak: Clonal Persistence in the Intensive Care Unit of a Brazilian Teaching Hospital

**DOI:** 10.3389/fmicb.2018.02997

**Published:** 2018-12-05

**Authors:** Danilo Yamamoto Thomaz, João Nobrega de Almeida, Glaucia Moreira Espindola Lima, Maína de Oliveira Nunes, Carlos Henrique Camargo, Rafaella de Carvalho Grenfell, Gil Benard, Gilda M. B. Del Negro

**Affiliations:** ^1^Laboratory of Medical Mycology—LIM-53, Clinical Dermatology Division, Hospital das Clínicas FMUSP and Instituto de Medicina Tropical de São Paulo, Universidade de São Paulo, São Paulo, Brazil; ^2^Central Laboratory Division—LIM-03, Hospital das Clínicas da Faculdade de Medicina da Universidade de São Paulo, São Paulo, Brazil; ^3^Laboratory of Clinical Analyzes, Hospital Universitário Maria Aparecida Pedrossian, Universidade Federal de Mato Grosso do Sul, Campo Grande, Brazil; ^4^Bacteriology Center, Instituto Adolfo Lutz, São Paulo, Brazil; ^5^Department of Biophysics, Escola Paulista de Medicina, Universidade Federal de São Paulo, São Paulo, Brazil

**Keywords:** *Candida parapsilosis*, candidemia, antifungal susceptibility, azole, resistance, *ERG11*, biofilm, genotyping

## Abstract

The incidence of candidemia by the *Candida parapsilosis* complex has increased considerably in recent decades, frequently related to use of indwelling intravascular catheters. The ability of this pathogen to colonize healthcare workers (HCW)' hands, and to form biofilm on medical devices has been associated with the occurrence of nosocomial outbreaks and high mortality rates. Fluconazole has been the leading antifungal drug for the treatment of invasive candidiasis in developing countries. However, azole-resistant *C. parapsilosis* isolates are emerging worldwide, including in Brazil. Few studies have correlated outbreak infections due to *C. parapsilosis* with virulence factors, such as biofilm production. We thus conducted a microbiological investigation of *C. parapsilosis* complex isolates from a Brazilian teaching hospital. Additionally, we identified a previously unrecognized outbreak caused by a persistent azole-resistant *C. parapsilosis* (*sensu stricto*) clone in the intensive care unit (ICU), correlating it with the main clinical data from the patients with invasive candidiasis. The molecular identification of the isolates was carried out by PCR-RFLP assay; antifungal susceptibility and biofilm formation were also evaluated. The genotyping of all *C. parapsilosis* (*sensu stricto*) was performed by microsatellite analysis and the presence of *ERG11* mutations was assessed in the azole non-susceptible isolates. Fourteen *C. parapsilosis* (*sensu stricto*) isolates were recovered from patients with invasive candidiasis, eight being fluconazole and voriconazole-resistant, and two intermediate only to fluconazole (FLC). All non-susceptible isolates showed a similar pattern of biofilm formation with low biomass and metabolic activity. The A395T mutation in *ERG11* was detected exclusively among the azole-resistant isolates. According to the microsatellite analysis, all azole non-susceptible isolates from the adult ICU were clustered together indicating the occurrence of an outbreak. Regarding clinical data, all patients infected by the clonal non-susceptible isolates and none of the patients infected by the susceptible isolates had been previously exposed to corticosteroids (p = 0.001), while the remaining characteristics showed no statistical significance. The current study revealed the persistence of an azole non-susceptible *C. parapsilosis* clone with low capacity to form biofilm over two years in the adult ICU. These results reinforce the need of epidemiological surveillance and monitoring antifungal susceptibility of *C. parapsilosis* isolates in hospital wards.

## Introduction

Hematogenous candidiasis (candidemia) is the most common presentation of invasive candidiasis (IC) in nosocomial settings, and is responsible for over 5% of all bloodstream infections (Arendrup, 2013; Cantey and Milstone, 2015). Candidemia causes increase in hospital length of stay and healthcare costs, and is associated with high mortality rates, usually over 40% in developing countries (Bloos et al., 2013; Antinori et al., 2016).

Although the incidence of candidemia by *C. albicans* is decreasing, it remains the most frequently isolated species in several centers, closely followed by *Candida glabrata, Candida tropicalis*, and *Candida parapsilosis*, all with the potential to exhibit resistance to fluconazole (FLC) and echinocandins (da Matta et al., 2017; Lamoth et al., 2018).

In 2005, based on the genetic diversity among *C. parapsilosis* isolates, two new cryptic species were described, and *C. parapsilosis* is now considered a species complex with *C. parapsilosis* (*sensu stricto*), *Candida orthopsilosis* and *Candida metapsilosis* (Tavanti et al., 2005). *Candida parapsilosis* is predominantly found in clinical specimens, while *C. orthopsilosis* and *C. metapsilosis* represent around 10% of isolates (Trofa et al., 2008; Nosek et al., 2009).

The incidence of *C. parapsilosis* fungemia has greatly increased over the last 30 years (Guinea, 2014), being the leading cause of candidemia in some European, Asian and Latin American medical centers (Singaravelu et al., 2014; Caggiano et al., 2017; da Matta et al., 2017). Neonates with invasive devices and echinocandin exposure have been related to *C. parapsilosis* bloodstream infections (Pammi et al., 2013).

The presence of *C. parapsilosis* on healthcare workers' (HCW) hands may contribute to horizontal transmission of this organism, causing invasive disease in patients with no prior evidence of colonization (Singaravelu et al., 2014). In addition, the ability of *C. parapsilosis* to form biofilm has been associated with colonization of medical devices, helping it to remain viable for at least 4 weeks on plastic healthcare surfaces, facilitating the occurrence of nosocomial outbreaks (Kuhn et al., 2004; Singaravelu et al., 2014; Welsh et al., 2017). After *C. albicans, C. parapsilosis* is the biggest biofilm producer among *Candida* species (Larkin et al., 2018) and candidemia by biofilm forming isolates has been associated with higher mortality rates (Tumbarello et al., 2007; Trofa et al., 2008).

*Candida parapsilosis* complex outbreaks have been reported worldwide since 2004, mainly in neonatal intensive care units (NICU), however the information regarding the susceptibility pattern of isolates has only been evaluated in a few studies (Wang et al., 2016; Benedict et al., 2017). The failure of infection prevention and control programs, such as inadequate environmental disinfection and hand hygiene, may be the main reason for the emergence of these outbreaks in hospital settings (Guo et al., 2015; Qi et al., 2018).

Although echinocandins are recommended as the initial therapy of IC in critically ill patients, FLC is an alternative drug for clinically stable patients with IC, and has been frequently administered as first choice antifungal agent (Bassetti et al., 2016; Pappas et al., 2016; O'Leary et al., 2017). Indeed, in developing countries, FLC is the leading antifungal for the treatment of IC, largely because of its lower price compared to echinocandins (Nucci et al., 2013a,b). However, this practice allied to biofilm production may be contributing factors for the concerning emergence of persistent clusters of FLC-resistant *C. parapsilosis*, as recently reported in Brazilian (Pinhati et al., 2016) and South African (Magobo et al., 2017) centers. In addition, inadequate antifungal treatment may increase the length of hospitalization and associated costs (Arnold et al., 2010).

In this study, a microbiological investigation of *C. parapsilosis* complex clinical isolates collected in a Brazilian public university hospital medical center between 2012 and 2016 was conducted. During this retrospective investigation, a previously unrecognized outbreak of a persistent azole-resistant *C. parapsilosis* (*sensu stricto*) clone was identified in the intensive care unit (ICU), correlating it with the main clinical data from the patients with IC.

## Materials and Methods

### Clinical Isolates

Seventeen clinical isolates of *C. parapsilosis* complex isolated at the University Hospital Maria Aparecida Pedrossian of the Federal University of Mato Grosso do Sul (HUMAP-UFMS) between April 2012 and March 2016 were studied. The institutional review board approved the study protocol (number 1.912.028). All organisms were previously identified by the VITEK® 2 system (bioMérieux, Marcy l'Etoile, France) in the HUMAP-UFMS and further analyses were performed in the Laboratory of Medical Mycology of the Institute of Tropical Medicine, University of São Paulo. Isolates from the same site of a given patient that were recovered at different times (time interval of ≤7 days) were excluded. Of the 17 *C. parapsilosis* complex isolates analyzed, 12 (70.6%) were recovered from blood cultures, four from central venous catheter (CVC) tips and one from bone marrow aspirate (BMA). Clinical characteristics of the patients are shown in Table 1.

**Table 1:** Identification and *in vitro* susceptibility testing of 17 *Candida parapsilosis* complex species from HUMAP-UFMS.

**Isolates**	**Clinical specimens**	**Species**	**Minimal Inhibitory Concentrations (mg/L)**				
			**AMB (S/I/R)**	**FLC (S/I/R)**	**VRC (S/I/R)**	**ANF (S/I/R)**	**MIF (S/I/R)**
16 PC	CVC tip	*C. parapsilosis*	1.0 (S)	64 (R)	1.0 (R)	2.0 (I)	1.0 (I)
58 H	Blood	*C. parapsilosis*	0.5 (S)	32 (R)	0.5 (R)	1.0 (I)	1.0 (I)
87 H	Blood	*C. parapsilosis*	0.5 (S)	64 (R)	2.0 (R)	2.0 (I)	1.0 (I)
88 H	Blood	*C. parapsilosis*	1.0 (S)	64 (R)	1.0 (R)	2.0 (I)	1.0 (I)
137 H	Blood	*C. parapsilosis*	0.5 (S)	64 (R)	1.0 (R)	2.0 (I)	1.0 (I)
542 AMO	BMA	*C. parapsilosis*	1.0 (S)	64 (R)	1.0 (R)	2.0 (I)	1.0 (I)
340 PC	CVC tip	*C. parapsilosis*	1.0 (S)	64 (R)	1.0 (R)	2.0 (I)	1.0 (I)
422 PC	CVC tip	*C. parapsilosis*	1.0 (S)	4.0 (I)	0.06 (S)	2.0 (I)	1.0 (I)
29 H	Blood	*C. parapsilosis*	0.5 (S)	4.0 (I)	0.125 (S)	2.0 (I)	1.0 (I)
119 H	Blood	*C. metapsilosis*	0.25 (S)	2.0 (S)	0.03 (S)	0.25 (I)	0.25 (I)
188 H	Blood	*C. parapsilosis*	0.5 (S)	1.0 (S)	0.03 (S)	1.0 (I)	1.0 (I)
199 H	Blood	*C. parapsilosis*	0.25 (S)	1.0 (S)	0.03 (S)	2.0 (I)	1.0 (I)
65 H	Blood	*C. orthopsilosis*	0.5 (S)	0.5 (S)	0.03 (S)	0.25 (I)	0.25 (I)
191 H	Blood	*C. orthopsilosis*	0.5 (S)	0.5 (S)	0.016 (S)	0.5 (I)	0.25 (I)
559 H	Blood	*C. parapsilosis*	0.5 (S)	2.0 (S)	0.06 (S)	1.0 (I)	0.5 (I)
1131 PC	CVC tip	*C. parapsilosis*	0.5 (S)	32 (R)	0.5 (R)	2.0 (I)	1.0 (I)
662 H-II	Blood	*C. parapsilosis*	0.06 (S)	0.5 (S)	0.008 (S)	0.5 (I)	0.5 (I)

*BMA, Bone marrow aspirate; CVC, Central venous catheter; AMB, Amphotericin B; ANF, Anidulafungin; CAF, Caspofungin; MIF, Micafungin; S, Susceptible; I, Intermediate; R, Resistant*.

### Identification of *C. parapsilosis* Complex Species

Initially, the identification of species was performed by matrix-assisted laser desorption ionization–time of flight mass spectrometry (MALDI-TOF MS) using Microflex mass spectrometer (Bruker Daltonics, Bremen, Germany). Enzymatic DNA extractions from the isolates were then carried out, following a previously described protocol (Van Burik et al., 1998). Molecular identification of the species was then achieved employing PCR-restriction fragment length polymorphism assay (RFLP) of the *SADH* gene (Tavanti et al., 2005) with *Ban*I enzyme (New England Biolabs, Ipswich, MA, USA).

### Antifungal Susceptibility Profile

Antifungal susceptibility testing was performed using the European Committee for Antimicrobial Susceptibility Testing (EUCAST) microdilution assay according to document E.DEF 7.3.1 (Arendrup et al., 2017). All isolates were tested for *in vitro* susceptibility to amphotericin B (Sigma-Aldrich, St. Louis, MO, USA), anidulafungin (Pfizer, New York, NY, USA), micafungin (Astellas Pharma, Tokyo, Japan), fluconazole (Sigma-Aldrich, St. Louis, MO, USA), and voriconazole (VRC, Sigma-Aldrich, St. Louis, MO, USA). The MICs were interpreted following the EUCAST clinical breakpoints (EUCAST, 2018). Each experiment was performed at least three times on different days, and *C. parapsilosis* ATCC 22019 and *C. krusei* ATCC 6258 were used as quality control strains.

### Biofilm Assay

Biofilm formation was carried out in 96-well microtiter plates (TPP, Trasadingen, Switzerland). Both biofilm biomass and metabolic activity were measured by using crystal violet (CV) staining (Melo et al., 2011) and XTT reduction assay (Pierce et al., 2008), respectively. The isolates were classified as low, moderate or high biofilm producers, as well as with low, moderate, or high metabolic activity, based on previously reported cutoff values (Marcos-Zambrano et al., 2014). Three independent experiments were performed and *C. albicans* SC5314, *C. parapsilosis* ATCC 22019, *C. orthopsilosis* ATCC 96141, and *C. metapsilosis* ATCC 96143 were used as quality control strains.

### Sequencing of the *ERG11* Gene

DNA samples from all *C. parapsilosis* (sensu stricto) isolates were submitted to both PCR and sequencing of the entire open reading frame (ORF) of the *ERG11* gene that encodes lanosterol 14 α-demethylase. PCR products were purified with the illustra™ ExoProStar™ 1-Step (GE Healthcare, Little Chalfont, Bucks, UK) and sequenced with the 3,500 Genetic Analyzer (Applied Biosystems, Foster City, CA, USA) using the four specific primers previously described (Souza et al., 2015). *ERG11* sequences were analyzed with the BioEdit v.7.2.3 sequence alignment editor (Hall, 1999) and compared with the available corresponding sequence of *C. parapsilosis* ATCC 22019 (GenBank accession no. GQ302972).

### Microsatellite Analysis

Genotyping of all *C. parapsilosis* (*sensu stricto*) clinical isolates and the reference strain ATCC 22019, was performed by microsatellite analysis using PCR amplification of eight different loci according to the procedures described by Pulcrano et al. (2012). PCR products were separated on 3% agarose gel, stained with GelRed™ (Biotium, Fremont, CA, USA) and visualized with UVITEC gel documentation system (Cleaver Scientific, Rugby, Warks, UK). The similarity of the allelic profiles was evaluated by the Dice coefficient and the clusters were analyzed with UPGMA employing the Bionumerics software v.7.5 (Applied Maths, Sint-Martens-Latem, Belgium).

### Clinical and Epidemiological Investigation

Epidemiological and clinical data, including demographic, underlying diseases, comorbidities, invasive procedures, previous exposure to antibiotics and antifungals were collected from patients with IC caused by *C. parapsilosis* (*sensu stricto*). Univariate analysis was carried out to assess possible risk factors for colonization/infection by azole non-susceptible *C. parapsilosis* (ANSCP) in relation to azole-susceptible *C. parapsilosis* (ASCP) isolates. Comparisons between groups were performed using Fisher's exact or chi-square tests, where appropriate, for the categorical variables. Continuous non-parametric variable comparison was done using the Mann-Whitney *U*-test. *P-*values of < 0.05 were considered statistically significant.

## Results

### Identification of *C. parapsilosis* Complex Species

Among the 17 *C. parapsilosis* complex isolates, 14 (82.4%) were identified as *C. parapsilosis* (*sensu stricto*), two as *C. orthopsilosis* and one as *C. metapsilosis* by both MALDI-TOF MS and RFLP assay (Table 1).

### Antifungal Susceptibility

All isolates were susceptible to amphotericin B and intermediate to anidulafungin and micafungin. Eight (57.1%) of 14 *C. parapsilosis* (*sensu stricto*) isolates were both FLC-resistant (MIC ≥32 mg/L) and VRC-resistant (MIC ≥0.5 mg/L), two isolates were intermediate (MIC = 4 mg/L) to FLC and the remaining *C. parapsilosis* (*sensu stricto*) isolates were susceptible. Both *C. orthopsilosis* and *C. metapsilosis* isolates were azole-susceptible (Table 1).

### Biofilm Formation

**Figure 1:**
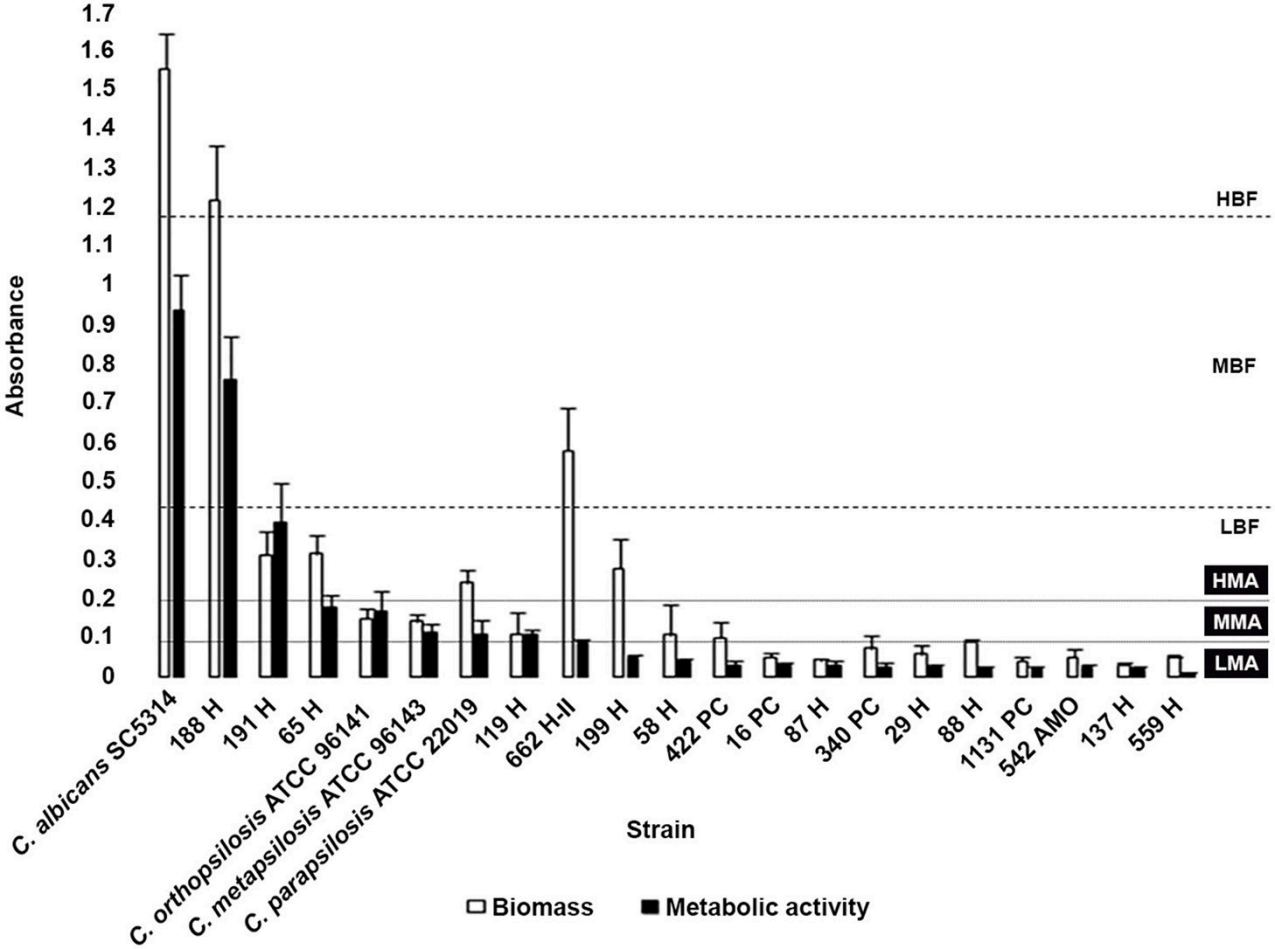
Comparison of biomass and metabolic activity of the biofilms formed by *Candida parapsilosis* complex reference strains and clinical isolates. Classification according to biomass production: LBF, Low biofilm-forming; MBF, Moderate biofilm-forming; and HBF, High biofilm-forming; and according to biofilm metabolic activity: LMA, Low metabolic activity; MMA, Moderate metabolicactivity; and HMA, High metabolic activity (Marcos-Zambrano et al., 2014). *Candida albicans* SC5314 reference strain was employed as biofilm formation control. Each result is representative of at least three experiments. Error bars represent standard deviation.

Biofilm formation was observed in all isolates (100%), with high variation between them. Biofilm metabolic activity was not correlated with the amount of biofilm biomass. **Two** *C. parapsilosis* isolates were classified as either high biofilm-forming (HBF) or moderate biofilm-forming. The HBF *C. parapsilosis* and one of the low biofilm-forming (LBF) *C. orthopsilosis* were classified as having biofilms with high metabolic activity; five isolates showed biofilms with moderate metabolic activity, including one *C. orthopsilosis* and the only *C. metapsilosis* isolate. The remaining ANSCP isolates showed a similar pattern of biofilm formation, classified as LBF with biofilms presenting low metabolic activity (Figure 1).

### Sequencing of the *ERG11* Gene

Analysis of the *ERG11* sequences of 1,569 bp in length revealed the presence of a homozygous silent mutation (T591C) in both the 10 ANSCP and 4 ASCP isolates. A missense mutation (A395T) that led to an Y132F amino acid substitution was observed in 7 (87.5%) of the 8 FLC and VRC-resistant isolates (Table 2). The G1193T mutation leading to R398I substitution was observed in only one ASCP isolate (188H).

**Table 2:** Susceptibility profile and *ERG11* sequence analysis of the 10 *Candida parapsilosis* (sensu stricto) isolates non-susceptible to azoles.

**Isolates**	**Isolation date**	**Hospital settings**	**Susceptibility profiles**		**Mutations in *ERG11* gene**
			**FLC**	**VRC**	
58 H	Apr/2012	Nephrology	Resistant	Resistant	T591C
87 H	Jun/2013	Adult ICU	Resistant	Resistant	T591C, A395T
137 H	Aug/2013	Adult ICU	Resistant	Resistant	T591C, A395T
88 H	Oct/2013	Adult ICU	Resistant	Resistant	T591C, A395T
542 AMO	Nov/2013	Adult ICU	Resistant	Resistant	T591C, A395T
16 PC	Jan/2014	Adult ICU	Resistant	Resistant	T591C, A395T
340 PC	Jul/2014	Adult ICU	Resistant	Resistant	T591C, A395T
422 PC	Aug/2014	Adult ICU	Intermediate	Susceptible	T591C
29 H	Jan/2015	Adult ICU	Intermediate	Susceptible	T591C
1131 PC	Sep/2015	Adult ICU	Resistant	Resistant	T591C, A395T

*ICU, Intensive care unit; FLC, Fluconazole; VRC, Voriconazole*.

### Clinical, Epidemiological, and Microsatellite Analysis

**Figure 2:**
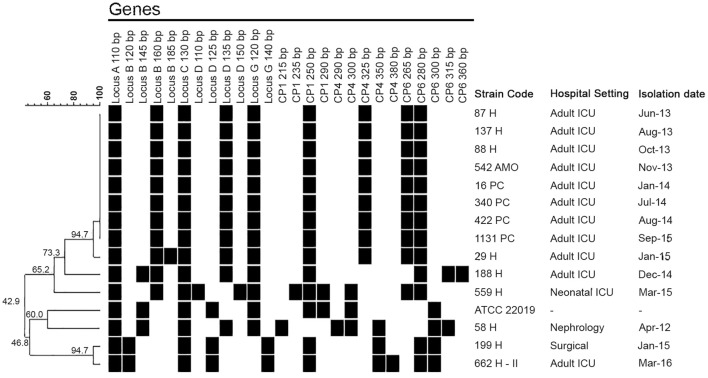
Dendrogram showing the clustering of the 14 *Candida parapsilosis* (*sensu stricto*) isolates and the ATCC 22019 strain based on microsatellite analysis. The black square indicates the presence of an amplification product.

Clinical and epidemiological data were collected from the records of the 14 patients with IC caused by *C. parapsilosis* (*sensu stricto*) isolates previously genotyped by microsatellite analysis. The first ANSCP isolate (58H) was detected in April 2012, recovered from an elderly patient with chronic renal failure undergoing hemodialysis via CVC. This patient had not been exposed to antifungal drugs during this hospitalization and developed candidemia in the nephrology unit. However, according to the microsatellite analysis, this isolate was not related to the other ANSCP (Figure 2).

Indeed, the first clonal ANSCP (87H) appeared in June 2013. A young adult patient with systemic lupus erythematosus undergoing prolonged hospitalization in the adult ICU developed breakthrough candidemia after 19 days of FLC treatment. After this case, other eight patients from the same unit showed positive cultures for ANSCP, totalizing four isolates from the blood, four from CVC and one from BMA (Tables 1, 2). According to the microsatellite analysis, all ANSCP isolates from the ICU were clustered. The final clone-related ANSCP (1131PC) isolate was detected in September 2015 (Figure 2).

The four ASCP isolates recovered between December 2014 and March 2016 were genetically unrelated to the outbreak clonal isolates. Patients were hospitalized in either the NICU (*n* = 1), the surgical ward (*n* = 1), or in adult ICU (*n* = 2) (Figure 2).

By comparing the clinical and epidemiological data retrieved from patients with clone-related ANSCP (*n* = 9) and from those with the unrelated ASCP isolates (*n* = 4), we found that patients infected by both groups of isolates were hospitalized for a prolonged period before showing positive *C. parapsilosis* culture (median 46 vs. 23.5 days, *p* = 0.3). Additionally, patients infected by clone-related ANSCP tended to have more comorbidities such as diabetes mellitus (44% vs. 0%) and chronic pulmonary diseases (89% vs. 25%), although these differences were not statistically significant. All patients with ANSCP isolates had been hospitalized in the adult ICU, while susceptible isolates infected only two patients from this unit (100% vs. 50%, *p* = 0.08). Most patients showed CVC prior positive cultures for *C. parapsilosis* (100% ANSCP vs. 75% ASCP). All patients infected by the clonal ANSCP had previous exposure to corticosteroids, while none of the patients with ASCP-associated infection had (*p* = 0.001). Most of the patients infected/colonized with clonal ANSCP had previous exposure to FLC (*n* = 7, 78%, range 2–67 days), but two patients did not receive FLC prior to showing positive cultures. Among the patients with ASCP*, one* (25%) also received FLC (*p* = 0.2). The median time of FLC exposure for the patients with the clonal ANSCP was higher than in the patients with ASCP (12 days vs. 0 days), but this was not statistically significant (*p* = 0.07, data not shown). The clinical and epidemiological data of both groups are summarized in Table 3.

**Table 3:** Epidemiological and clinical characteristics of HUMAP-UFMS patients with positive cultures for clonal azole non-susceptible (ANSCP) and azole-susceptible (ASCP) *Candida parapsilosis* isolates.

	**ANSCP (*n* = 9)**	**ASCP (*n* = 4)**	***P-*value**
Age (years)—median (range)	41 (22–84)	28 (1–45)	0.2
Gender female—n (%)	5 (56)	3 (75)	1
Days of hospitalization before positive culture	46 (1–106)	23.5 (10–92)	0.3
Apache II score—median (range)^*^	20.3 (8–35)	12 (12)	NA
SOFA score—median (range)^**^	6.4 (3–12)	9.5 (1–18)	1
Prior adult ICU admission—*n* (%)	9 (100)	2 (50)	0.08
**Comorbidities**—***n*** **(%)**			
Cancer	0 (0)	1 (25)	0.3
Pulmonary disease	8 (89)	1 (25)	0.09
Cardiac disease	2 (22)	1 (25)	1
Diabetes mellitus	4 (44)	0 (0)	0.2
Renal failure	5 (56)	1 (25)	0.5
Hepatic failure	2 (22)	0 (0)	1
**Previous Exposure To Invasive Procedures—*****n*** **(%)**			
Urinary catheter	8 (89)	2 (50)	0.2
Central venous catheter	9 (100)	3 (75)	0.3
Previous hemodialysis	4 (44)	0 (0)	0.2
Mechanical ventilation	9 (100)	3 (75)	0.3
Previous antibiotic exposure	9 (100)	4 (100)	1
Previous corticosteroid exposure	9 (100)	0 (0)	**0.001**
Previous antifungal exposure	9 (100)	2 (50)	0.07
Previous fluconazole exposure	7 (78)	1 (25)	0.2
**Previous** ***Candida*** **Colonization**			
*Candida parapsilosis*	5 (56)	1 (25)	0.5
Other species	9 (100)	2 (50)	0.07
30-days all cause mortality	7 (78)	2 (50)	0.5

* Data missing in five patients

** Data missing in four patients *NA, Not applicable due to insufficient number of patients*. *The P-value in bold means that it was statistically significant*.

Of the 10 patients with proven candidemia, 8 were treated with echinocandin-based regimen; of these, seven (87.5%) died. Detailed data of these patients are shown in Table 4.

**Table 4:** Clinical and epidemiological data from the 10 cases of proven *Candida parapsilosis* (*sensu stricto*) fungemia at HUMAP-UFMS.

	**Patient data**								
**Isolate**	**Underlying conditions**	**Days of hospitalization before candidemia**	**Central venous catheter at candidemia**	**Previous antibiotic exposure**	**Previous steroid exposure**	**Previous fluconazol and/or echinocandin exposure**	**Previous *Candida* colonization**	**Antifungal treatment**	**30-days outcome**
58 H	Elderly patient, pneumonia, renal, and hepatic failure, diabetes mellitus, septic arthritis	1	Yes	No	No	No	Yes	ANF	Dead
87 H^*^	SLE, peritonitis abdominal surgery	21	Yes	Yes	Yes	FLC/MIF	Yes	MIF	Dead
137 H^*^	Elderly patient, pneumonia, infected pressure ulcer, renal failure	106	Yes	Yes	Yes	FLC	Yes	MIF	Dead
88 H^*^	Pulmonary PCM, acute respiratory failure	39	Yes	Yes	Yes	FLC	Yes	ANF	Alive
542 AMO^*^	Elderly patient, COPD, acute myocardial infarction, heart failure, ventilation-associated pneumonia	81	Yes	Yes	Yes	FLC/MIF	Yes	MIF	Dead
29 H^*^	HIV-associated pneumocystosis, renal failure, inflammatory bowel disease	25	Yes	Yes	Yes	FLC/ANF	Yes	ANF	Dead
188 H	Cerebral palsy, repeated pneumonia, intestinal obstruction	92	Yes	Yes	No	ANF	Yes	ANF	Dead
199 H	Renal abscess, renal failure, sickle cell disease	23	No	Yes	No	No	No	No	Alive
559 H	Prematurity, bacterial sepsis, mechanical ventilation, parenteral nutrition	24	Yes	Yes	No	No	No	AMB	Alive
662 H-II	Acute peritonitis, pelvic abscess, gastric adenocarcinoma, arrhythmia	10	Yes	Yes	No	FLC	Yes	MIF	Dead

* *Clonal isolates*. *COPD, Chronic obstructive pulmonary disease; PCM, Paracoccidioidomycosis; SLE, Systemic lupus erythematosus; FLC, Fluconazole; MIF, Micafungin; ANF, Anidulafungin; AMB, Amphotericin B*.

## Discussion

Some studies have shown that ANSCP isolates tend to cluster in ICUs (Pfaller et al., 2008; Raghuram et al., 2012; Govender et al., 2016), although the molecular analysis of the isolates was not performed in these reports. Magobo et al. (2017) reported previously undetected outbreaks in NICU of FLC-resistant *C. parapsilosis* in a South African hospital in a retrospective study employing molecular typing (Magobo et al., 2017).

The high number of cases of ANSCP (71.4%) observed in this cohort, led to a molecular analysis of these isolates to reveal the occurrence of probable outbreak in the adult ICU. This study revealed the persistence of an azole non-susceptible *C. parapsilosis* clone over 2 years in this unit. Indeed, although most *C. parapsilosis* complex isolates are usually susceptible to azoles, recent Brazilian reports have indicated the emergence of IC due to ANSCP (da Costa et al., 2014; Giacobino et al., 2016; Alencar et al., 2017). To our knowledge, this is the second reported outbreak of ANSCP in Brazil (Pinhati et al., 2016).

Low resistance rates to FLC have been reported among *C. parapsilosis* isolates worldwide, including Latin America (1.1%) (Nucci et al., 2013a), USA (4.0%) (Pfaller and Castanheira, 2016), and Asia-Western Pacific (5.7%) (Pfaller et al., 2015). However, undetected outbreaks of ANSCP infection could be ongoing in Brazil and other countries because at present there are very few studies aiming to continuously monitor azole resistance in this species and investigate the molecular relationship between isolates (Pinhati et al., 2016; Magobo et al., 2017).

Recent reports indicate that mutations in *ERG11* gene contribute to azole resistance in *C. parapsilosis* (Berkow et al., 2015; Grossman et al., 2015; Asadzadeh et al., 2017). In accordance with this finding, we observed that the A395T mutation in *ERG11* occurs exclusively among azole-resistant isolates. However, in one FLC-resistant isolate (58H) this mutation was not observed, indicating that azole resistance involves other molecular mechanisms, such as overexpression of *ERG11* and efflux pumps (Souza et al., 2015). Further analysis of overexpression of *ERG11, CDR1*, and *MDR1* should be performed to clarify the non-susceptibility of isolates without missense mutation in *ERG11*.

The high prevalence of the A395T mutation in FLC-resistant *C. parapsilosis* clinical isolates was also noted in previous studies by Grossman et al. (2015) and Choi et al. (2018). In addition, the latter suggested that isolates with this mutation may have a higher propensity to cause clonal transmission and to persist in nosocomial settings than FLC-resistant *C. parapsilosis* without A395T mutation. Regarding our ASCP isolates, the G1193T mutation was detected in only one isolate (188H). However, other studies identified this mutation in both susceptible and resistant isolates, suggesting that this mutation alone is not related to azole resistance (Berkow et al., 2015; Grossman et al., 2015; Asadzadeh et al., 2017).

Kuhn et al. correlated an outbreak of *C. parapsilosis* infections with virulence factors, and they observed that clonal isolates had a higher ability to form biofilms than unrelated strains, suggesting that biofilm production plays a role in *C. parapsilosis* outbreaks (Kuhn et al., 2004). By contrast, all clonal isolates of our study displayed a low capacity to form biofilm, indicating that this ability may not be a critical causal factor of outbreaks.

Wang et al. performed phylogenetic analyses of *C. parapsilosis* isolates from concomitant blood and CVC tip cultures that show identical genotypes, indicating that the catheter is an important source of infection by this species (Wang et al., 2016). Moreover, several studies have demonstrated horizontal transmission in IC outbreaks by *C. parapsilosis*, illustrating the genetic similarities between clinical and isolates found on HCW hands (Huang et al., 1999; Barchiesi et al., 2004; van Asbeck et al., 2007; Hernández-Castro et al., 2010). In our cohort, four clonal ANSCP were recovered from CVC tips indicating the inadequate handling of this device as a probable route of transmission of this outbreak.

Our molecular epidemiological investigation revealed that all clonal ANSCP isolates were detected in the adult ICU (Figure 2). One of the related isolates (29H) displayed an exclusive heterozygosis in Locus B, which may indicate a possible genetic microevolution that may be related to stress conditions, such as previous antifungal exposure (Sabino et al., 2010; Pulcrano et al., 2012). Although FLC-resistant, the 58H isolate presented low genetic similarity (42.9%) to the ANSCP clone (Figure 2). We believe that the geographic distance of the nephrology unit from the adult ICU, as well as the time that elapsed between the isolation of the 58H and the clonal ANSCP isolates (over 1 year) may explain the lack of relation between them.

Pinhati et al. (2016) found that diabetes was an independent risk factor for infection by ANSCP. They concluded that diabetic patients are more prone to be colonized by *Candida*, as this medical condition is associated with increased contact between HCW and patients (Pinhati et al., 2016). A similar pattern was found in this study, with 44 and 0% of the patients with ANSCP and ASCP isolates, respectively, having diabetes. However, due to the low number of patients analyzed, the difference between the groups was not statistically significant. There was a trend (*p* = 0.07) for previous antifungal exposure and positive ANSCP cultures. Three of the patients with ANSCP did not receive FLC for over 48 h, which may explain the probable horizontal transmission between the patients (data not shown). We found that having received corticosteroids was associated with positive culture for the clonal ANSCP (Table 3). Indeed, most of the patients that were admitted to the adult ICU had pulmonary diseases requiring corticosteroid prescription. We believe that the persistence of the clonal ANSCP isolates may have been due to contaminated instruments, environment and HCW's hands. Unfortunately, as this study was performed retrospectively, samples from neither HCW hands nor the environment could be collected.

Echinocandins have been recommended as first-line treatment for candidemia by the main clinical guidelines (Cornely et al., 2012; Pappas et al., 2016), including fungemia by *C. parapsilosis*. Indeed, similar 30-days mortality rates have been observed between echinocandin- and fluconazole-treated patients with fungemia by this species (Fernández-Ruiz et al., 2014; Chiotos et al., 2016). On the other hand, *C. parapsilosis* isolates demonstrate innately high MICs for echinocandins (Garcia-Effron et al., 2008), which led to EUCAST to classify isolates with MICs below 2 mg/L as intermediate, instead of susceptible as recommended by the CLSI (Meletiadis et al., 2016). Most of our patients with candidemia were treated with echinocandins and 87.5% showed poor outcome. However, at least 60% of these patients had severe comorbidities (Table 4) that could have contributed more importantly to lethality (Lin et al., 2015).

In conclusion, our results suggest the possibility of persistence of clonally related azole-resistant *C. parapsilosis* (*sensu stricto*) isolates in hospital settings, irrespective of their capacity of biofilm formation. Furthermore, azole resistance in *C. parapsilosis* isolates is emerging in Brazilian medical centers, and the molecular mechanisms involved should be monitored in conjunction with azole susceptibility profiles. Finally, the screening of additional samples from HCW hands, indwelling devices and environmental surfaces will improve understanding of the nosocomial transmission process, and will help to control the spread of ANSCP clones more effectively.

## Ethics Statement

This study was carried out in accordance with the recommendations of the research ethics committee of the Medical School of the University of São Paulo, São Paulo, Brazil. The protocol number 1.912.028 was approved by the research ethics committee of the Medical School of the University of São Paulo for analysis of microorganisms and clinical data records, without the need for written informed consent of the subjects.

## Author Contributions

DT and GD designed the study, helped with acquisition and data analysis, drafted and revised the work, approved the final work, and agreed with all the aspects of the work. JA helped with acquisition and data analysis, drafted and revised the work, approved the final work, and agreed with all the aspects of the work. GL and MN helped with acquisition of the data, approved the final work, and agreed with all the aspects of the work. CC and RG helped with data analysis, approved the final work, and agreed with all the aspects of the work. GB helped with data analysis, drafted and revised the work, approved the final work, and agreed with all the aspects of the work.

### Conflict of Interest Statement

The authors declare that the research was conducted in the absence of any commercial or financial relationships that could be construed as a potential conflict of interest.
